# Supplementary material for: The impact of P-element-induced hybrid dysgenesis on the male germline in Drosophila simulans
Source: Genetics. 2026 Aug 20;234(2):iyag219. doi: 10.1093/genetics/iyag219 (PMC13643802; doi:10.1093/genetics/iyag219)
Supplement: Supplementary file 1 [file iyag219_peer_review_history.pdf]

GENETICS

The impact of P-Element-induced hybrid dysgenesis on the male germ-line in Drosophila simulans

Joanne Griffin, Ewan Harney, Charlton Capes, Rowan Connell, Andrea Betancourt, and Valeria Romero-Soriano

NOTE: The reviews and decision letters are unedited and appear as submitted by the reviewers.
In extremely rare instances and as determined by a Senior Editor or the EIC, portions of a review may be redacted. If a review is signed, the reviewer has agreed to no longer remain anonymous.
The review history appears in chronological order.

Review Timeline:

Submission Date:	2025-10-30
Editorial Decision:	2026-01-17
Resubmission Received:	2026-06-26
Accepted:	2026-07-25

1st Editorial Decision: January 17, 2026

January 16, 2026

GENETICS-2025-308691 The impact of P-Element-induced hybrid dysgenesis on the male germ-line in Drosophila simulans

Dear Dr. Betancourt:

Two experts in the field have reviewed your manuscript, and I have read it as well. These reviewers had rather different opinions on your manuscript, with reviewer 1 being quite positive about it while reviewer 2 was considerably more negative. From my own reading, I do think that your manuscript has value to offer the field, as hybrid dysgenesis in males has been under-studied in comparison to that in females. This would argue for its acceptance; however, I also agree with the reviewers that some parts of the paper were hard to follow. So I think the best course of action would be for us to reconsider the manuscript once revisions have been made to address these criticisms. This should result in a stronger and more impactful paper.

When revising this manuscript, please make sure to address Reviewer 2's comment that alternative splicing can have an important effect on P element regulation. You should also carefully consider the comments and suggestions from both reviewers about ways to present your data and arguments with more clarity.

We look forward to receiving your revised manuscript. Please let the editorial office know approximately how long you expect to need for revisions.

Upon resubmission, please include:

A clean version of your manuscript;

A marked version of your manuscript in which you highlight significant revisions carried out in response to the major points raised by the editor/reviewers (track changes is acceptable if preferred);

A detailed response to the editor's/reviewers' comments and to the concerns listed above. Please reference line numbers in this response to aid the editors.

Additionally, please ensure that your resubmission is formatted for GENETICS.
https://academic.oup.com/genetics/pages/general-instructions

Follow this link to submit the revised manuscript: Link Not Available

Sincerely,

William Gilliland Associate Editor GENETICS

Approved by: Amy MacQueen Senior Editor GENETICS

Reviewer #1 :

This is a very nice contribution to our understanding of the impacts of P element induced dysgenesis in males. This is an area of investigation that has been somewhat ignored since the original investigations of dysgenesis in the 70's and 80s and this provides some important new insight into how the male gonad responds to excess activation by the P element.

I have no issues with the conclusions. My recommendations are motivated to provide clarity for the reader.

Line 50...purged by natural selection.

Line 255...I am not sure they are doing their statistics the best way. My understanding is that dysgenesis is sort of an all or nothing thing for a given testis. Either the germline stem cells are all lost, or, if not, even a single germe cell on one side can basically divide sufficiently to restore the germline stem cell population. So, the question is, whether a given testis is fully ablated or not. If one testis is ablated, the other one is probably sufficient to produce large numbers of sperm and produce no noticeable effect. I noticed from figure 2, that four dysgenic males are completely sterile, and none from the reciprocal cross. If you do a Fisher's exact, with one-tail (that seems reasonable in this case) 4:22::0:30, the p-value is 0.0407. Not an extremely strong result (and they should check my math), but it seems worth presenting this. The key point here is that if the germline stem cells escape utter death, I think there might nor be a strong effect once they have repopulated the niche.

Line 263...were any of the testes fully atrophied? Were they included in the RNA-seq

Line 269..It might be worth also showing a PCA with just the reciprocal progeny (dysgenic, non-dysgenic both young and old). PC1 seems to be driven by genetics with, as you might expect, the hybrids mostly in the middle. If you did a PCA with out those parental samples, you could see partitioning perhaps entirely driven by the maternal effect and dysgenesis. I would recommend adding that to Supplemental 2

Line 350.. And figure 8. Even if not significant, they should point out the abundance of the P element piRNA in. I also think that, in addition to the volcano plots, they should plot piRNA abundance of each TE in each direction of the cross against eachother. For example, similar to figure 2 in Brennecke et al, 2008

Line 390 - Figure 9B is really neat!

Line 420 - For clarity, maybe in the parentheses, revise to:

(Backcross (BC) progeny from F1 females: n = 51 insertions in 488 BC progeny from reciprocal F1 females and 307 insertions in 472 BC progeny from dysgenic F1 femalesfl, F1 males: 37 in 570 BC progeny from reciprocal F1 males and 271 in 566 BC progeny from dysgenic F1 males flies).

Figure 5. Even if not significant, there should be some arrow or something that identifies P element expression in 5B and 5C. Likewise for figure 8.

Figure 9A is interesting, but it would be worth also presenting parsed by the 8 conditions (4 samples and 2 ages). Otherwise, it is difficult to determine if this pattern is consistent

Reviewer #2 :

Overall I'm neutral on this manuscript. The majority of the results seem to be inconclusive and presented in a way that's confusing. With all the different comparisons it was hard to keep track of what was 'expected' based on the cross, and then what the result was. I think the manuscript would benefit from a lot more summary tables and/or figures to more clearly delineate the implications of the results.

I don't necessarily have a problem with presenting negative results, but they should be more clearly described as such. In addition, there was no mention of the regulation of the P-element by alternative splicing, or discussion of how inheritance of the Y from a mother and X from a father could affect the epigenetics of the chromosome. Even if its known to have no effect, just to see that the authors considered it.

I have lots of small notes about grammatical mistakes and use of acronyms below.

Abstract - a little unclear in how it is organized, P-element is introduced then we move to general information about TEs then back to the P-element. Would maybe keep it general then focus. One sentence emphasizing the importance of the P-element. Lots of use of punctuation to make sentences needlessly long. Uses an acronym for TE without introducing it.

47 - make two sentences

57 - LTR and DNA are never introduced. You provide the basics of what a TE is, but not what these are.

59 - this sentence is five lines long

77 - I don't see how it raises the possibility that it spreads more easily, especially considering the F1 phenotype more severely affects females. Maybe just raises the possibility that the P-element also spreads in the male germline

In general the smattering of citations within sentences and also at the end of the sentences I think is interfering with the authors ability to see when they need commas or other punctuation. Just for ease I would suggest putting them at the end of a sentence. They are often put where a common would be in the middle of a sentence with no comma, it makes it difficult to read.

80 some kind of missing punctuation

95 what is the origin of M252

100 - HD was never defined as an acronym

100 - what is a P-type line and an M-type line

100 - I feel like this would really benefit from an illustration. I want to see how the crosses work with the actual chromosomes drawn out. 125 - I can't tell if this is a biological or technical replicate. Why only two replicates? RNA-seq is so cheap. You can't estimate variance with only two.

142 - what is a decoy aware index

150 - In general I don't know why we are doing GO analyses other than that we can. I wouldn't define acronyms here since you aren't going to use them for a long time

214 - what is a small pool

225 - from the description its difficult to tell what was done here. Like, what does multiinter do? What do you mean they were combined? Its just a little vague.

P-element is italicized here and not elsewhere in the manuscript.

I don't think it is appropriate to be removing PCR duplicates here? If you can explain why you would do that when its not generally done for RNA-seq.

Did the methods section used to be after the results section? Figure 1 is what I was looking for in the methods section but its not referenced there, only seeing it in the results. However, the figure is blurry and still a little unclear. Like, what do the blue and red bubbles mean if they aren't used in both figures? If you are indicating P-cytotype with the red bubble, shouldn't it be red on the left hand side too? Or are you trying to indicate something else?

Its weird that the Drosophila/D. contraction is introduced over and over.

By dysgenic you mean not suppressed right? You're calling it the phenotype before you've established it has the phenotype.

Is it possible that epigenetic differences resulting from maternal inheritance of the Y could be causing these differences? Can you explain why that's not a possibility? Like normally if mom contributes the X you would have mom effects on it, but with the Y? I know Drosophila does not have DNA methylation, but there are plenty of other epigenetic effects.

258 - I feel like no difference in total offspring production is pretty important.....

266 - why define an acronym you never use

271 - you're calling it the 'effect of dysgenesis' but is it, if they produced the same number of offspring overall? 271 -283 - hard to digest this paragraph. How much do these lists overlap? Are the same genes disrupted in any comparisons? This is mentioned once but it's a lot of lists of numbers.

288 - you defined acronyms in the methods for Molecular Function, biological process etc, but here you are spelling them out. I don't suggest using the acronyms by any means. 285 - 296 -what have we learned or gained from this analysis? Anything? I would argue no.

302 - this paragraph seems pretty important. So if you lump old and young together, the expression of the P-element is different, but if you separate them out you no longer have the power to say it is different? Do you have outliers that are reducing your power by a lot? Non-target TEs are significantly differently expressed though. I'm not overly convinced by this analysis so far. I'm confused, maybe I'm misinterpreting what you did. Having the axes all the same might help. In Figure A, its confusing that the P-element would be so close to a log2fold change of maybe 1? But so significant. Is there a better way to illustrate the expression and variance in expression of the elements of interest?

315 - so the p-element is differentially expressed in the cross where one might expect it not to change, but not between the crosses where one might expect it to change? Am I getting these results mixed up? These are a lot of comparisons and genotypes to keep track of, I don't know if there is a clearer way to illustrate the results. Maybe a table.

369 - Ok it seems like this paper is mostly reporting negative results unless I'm mistaken. I don't have an issue with reporting negative results, but more clearly presenting them as such. might be worthwhile. 389 - P-element cognate piRNAs and a decrease in P-element mRNA levels - did I miss this being reported? Is this significant? Is there a figure?

391 - We were also interested in whether TE mRNA and piRNA production from F1 flies resembled an intermediate value between the two parents, or that of the maternal parent Which was it? 401 In contrast to dysgenic F1 flies, there are no large disparities between Shellder mRNA and cognate small RNAs, suggesting that Shellder is well-regulated compared to the P-element (Supplementary Figure 5C). On what basis are we saying that the P-element isn't well regulated?

436 - I don't understand the caveat? Do you mean there was dysgenesis in flies that didn't differ in p-element status?

None of this considers the fact that the P-element has considerable evidence for regulation by alternative splicing as well as piRNA. I know this is often disregarded in manuscripts on the P-element. "Unexpectedly, we show that the piRNA pathway components do not act to reduce transcript levels of the P-element transposon during P-M hybrid dysgenesis, a syndrome that affects germline development in Drosophila. Instead, splicing regulation is mechanistically achieved together with piRNA-mediated changes to repressive chromatin states, and relies on the function of the Piwi-piRNA complex proteins Asterix (also known as Gtsf1) and Panoramix (Silencio), as well as Heterochromatin protein 1a (HP1a; encoded by Su(var)205). "

Associate Editor Comments:

I thought your use of a compound-X in your crossing scheme, producing males with identical chromosomal genotypes but different cytotypes, was a very clever approach. However, it does mean that the parent-of-origin for the sex chromosomes is reversed (e.g. the Y is matroclinous in the cross with a compound-X mother, and patroclinous in the cross with a normal-X mother.) Do you think that this parent of origin difference could lead to epigenetic changes that might be having an effect? I recognize that this difference would also be confounded with the cytotype differences in this particular cross, but a brief discussion of this point (with possible experiments that might tease these apart) would be welcome.

Please find attached a new version of our manuscript on P-element dysgenesis in *Drosophila simulans* males. We have substantially revised the manuscript, including restructuring the results and adding new analyses which we think better capture the biology of P-element dysgenesis. We thank the reviewers and the associate editor for their comments, which have greatly helped in improving the manuscript. We answer the reviewer comments point-by-point below:

Reviewer #1:

This is a very nice contribution to our understanding of the impacts of P element induced dysgenesis in males. This is an area of investigation that has been somewhat ignored since the original investigations of dysgenesis in the 70's and 80s and this provides some important new insight into how the male gonad responds to excess activation by the P element.

Thank you for the kind words and very helpful comments.

I have no issues with the conclusions. My recommendations are motivated to provide clarity for the reader.

1. Line 50...purged by natural selection.

Changed to 'lost'—as copies of TEs can be lost by genetic drift or sometimes by excision (line 61).

2. Line 255...I am not sure they are doing their statistics the best way. My understanding is that dysgenesis is sort of an all or nothing thing for a given testis. Either the germline stem cells are all lost, or, if not, even a single germe cell on one side can basically divide sufficiently to restore the germline stem cell population. So, the question is, whether a given testis is fully ablated or not. If one testis is ablated, the other one is probably sufficient to produce large numbers of sperm and produce no noticeable effect.
I noticed from figure 2, that four dysgenic males are completely sterile, and none from the reciprocal cross. If you do a Fisher's exact, with one-tail (that seems reasonable in this case) 4:22::0:30, the p-value is 0.0407. Not an extremely strong result (and they should check my math), but it seems worth presenting this. The key point here is that if the germline stem cells escape utter death, I think there might nor be a strong effect once they have repopulated the niche.

Thanks for this suggestion! Indeed, the reviewer's math is correct, and we should have noticed this before, especially as it parallels an earlier analysis we did for females. We now include this analysis around lines 456. We also compare more directly to previous results for females (please see lines 738 and on).

3. Line 263...were any of the testes fully atrophied? Were they included in the RNA-seq
While we didn't dissect the males used for the fertility assays, we did note gonadal atrophy on testes collected from the dissections used for RNA-seq. We didn't collect this data systematically enough to include it as a result in the manuscript, but we did observe some level of gonadal atrophy in F1 males – some flies had one or both testes reduced in size, and some had also one or two fully atrophic testes. The proportion of affected testes recorded is fewer 1 in 10, roughly consistent with the proportion of fully sterile males.

4. Line 269..It might be worth also showing a PCA with just the reciprocal progeny (dysgenic, non-dysgenic both young and old). PC1 seems to be driven by genetics with, as you might expect, the hybrids mostly in the middle. If you did a PCA with out those parental samples, you could see partitioning perhaps entirely driven by the maternal effect and dysgenesis. I would recommend adding that to Supplemental 2

This is an interesting thought, but we feel it replicates the differential expression analysis in a slightly non-standard way. With this plot, we were only attempting the standard quality control done for RNAseq experiments generally.

5. Line 350.. And figure 8. Even if not significant, they should point out the abundance of the P element piRNA in.

We have added labels for the P-element in all relevant volcano plots.

I also think that, in addition to the volcano plots, they should plot piRNA abundance of each TE in each direction of the cross against each other. For example, similar to figure 2 in Brennecke et al, 2008

We have added these plots to Figures 2 and 8.

6. Line 390 - Figure 9B is really neat! :)

:)

7. Line 420 - For clarity, maybe in the parentheses, revise to:
(Backcross (BC) progeny from F1 females: n = 51 insertions in 488 BC progeny from reciprocal F1 females and 307 insertions in 472 BC progeny from dysgenic F1 femalesfl, F1 males: 37 in 570 BC progeny from reciprocal F1 males and 271 in 566 BC progeny from dysgenic F1 males flies).

In the end, we decided to incorporate these numbers into the relevant figure, as clear phrasing of all these numbers and types seemed to be beyond us. Please see revised figure 3.

Figure 5. Even if not significant, there should be some arrow or something that identifies P element expression in 5B and 5C. Likewise for figure 8.
We have added labels for the P-element in all relevant volcano plots.

Figure 9A is interesting, but it would be worth also presenting parsed by the 8 conditions (4 samples and 2 ages). Otherwise, it is difficult to determine if this pattern is consistent

We see the reviewer's point but addressed it by taking a slightly different tack—we analysed all of the data together using a linear model, and show that sample type and age do significantly but slightly affect significantly the slope of the relationship. However, the main point is that the relationship is positive, which is not unexpected as small RNAs are mainly generated from the TE transcripts.
We now include these results in the figure legend (see Figure legend 8) and deemphasize the information from individual sample types by fitting a single line for illustration purposes. We also excluded TEs/piRNAs with no expression to better visualise the results (and as it artificially elevates the sample size to show that TEs that are not there have no TE and no piRNA expression).

Reviewer #2 :

Overall I'm neutral on this manuscript. The majority of the results seem to be inconclusive and presented in a way that's confusing. With all the different comparisons it was hard to keep track of what was 'expected' based on the cross, and then what the result was. I think the manuscript would benefit from a lot more summary tables and/or figures to more clearly delineate the implications of the results.

I don't necessarily have a problem with presenting negative results, but they should be more clearly described as such.

In addition, there was no mention of the regulation of the P-element by alternative splicing, or discussion of how inheritance of the Y from a mother and X from a father could affect the epigenetics of the chromosome. Even if its known to have no effect, just to see that the authors considered it.

Despite the reviewer's overall lack of enthusiasm, there are a great many helpful comments in the below which we have tried to address. Moreover, we can see from the reviewer's comments below where we did not clearly convey aspects of our study (the unusual stream-of-consciousness style was actually quite helpful in this regard!)

We have also substantially reorganised the manuscript, which hopefully highlights the non-negative results and the rationale behind each aspect a little better.

========================

I have lots of small notes about grammatical mistakes and use of acronyms below.

Abstract - a little unclear in how it is organized, P-element is introduced then we move to general information about TEs then back to the P-element. Would maybe keep it general then focus. One sentence emphasizing the

importance of the P-element. Lots of use of punctuation to make sentences needlessly long. Uses an acronym for TE without introducing it.
The abstract has been restructured – hopefully it is clear now.

47 - make two sentences
We have now split this sentence in two.

57 - LTR and DNA are never introduced. You provide the basics of what a TE is, but not what these are.
Thanks for this - we have removed this bit for simplicity.

59 - this sentence is five lines long
We have now split this first sentence in two.

77- I don't see how it raises the possibility that it spreads more easily, especially considering the F1 phenotype more severely affects females. Maybe just raises the possibility that the P-element also spreads in the male germline
We have rephrased this for clarity. The principle is that the dysgenic phenotype is more severe in females, so females are more commonly sterile / less fertile. Even if the effect is quantitative, the "lower loss of fertility" in males might mean more of their offspring will inherit new P-element insertions, which will contribute to the spread of the TE through generations.

In general the smattering of citations within sentences and also at the end of the sentences I think is interfering with the authors ability to see when they need commas or other punctuation. Just for ease I would suggest putting them at the end of a sentence. They are often put where a common would be in the middle of a sentence with no comma, it makes it difficult to read.

We have split longer sentences throughout the introduction, which we hope improves the readability of the manuscript.

80 some kind of missing punctuation
Oxford comma added.

95 what is the origin of M252

Madagascar; now included on line 102.

100 - HD was never defined as an acronym
Thanks - we have restructured this paragraph for clarity, the acronym isn't there anymore.

100 - what is a P-type line and an M-type line
These concepts are now introduced in at lines 104 and on in the Methods, and again around line 307 in the Result for those readers who skip the Methods.

========================

100 - I feel like this would really benefit from an illustration. I want to see how the crosses work with the actual chromosomes drawn out.

We have revised this figure, and hope it is more useful. We now cite it in both the methods and the results section (ca line 105 and 312).

125 - I can't tell if this is a biological or technical replicate. Why only two replicates? RNA-seq is so cheap. You can't estimate variance with only two.

We have clarified at line 143-4 that these are biological replicates.
We do note that we have 4 replicates for dysgenic vs. reciprocal comparisons, and include age as a covariant in those comparisons. We do try to be appropriately cautious about strong conclusions based on the two replicate comparisons only, and have de-emphasised these results (e.g., moving the related figures to Supplementary Information). (Also note that this work was primarily funded by an MSCA fellowship grant to VRS, which did not have a large budget for sequencing.)

142 - what is a decoy aware index

This is a standard part of the Salmon pipeline; we reduced the text here as these details were unnecessary.

150 - In general I don't know why we are doing GO analyses other than that we can. I wouldn't define acronyms here since you aren't going to use them for a long time
We shortened the description here and lost the acryonyms (see lines ca 172). We have also tried to clarify the motivations in the paragraph starting at line 420.
We have also added an analysis of the enrichment of cell types showing a possible loss of mature sperm cells, which we think is more enlightening (see paragraph starting line 434).

214 - what is a small pool
We have clarified that these are the 'individual pools of eight flies' (line 268).

225 - from the description its difficult to tell what was done here. Like, what does multiinter do? What do you mean they were combined? Its just a little vague.

We have revised to "To identify insertions that were unique (and thus potentially represent new insertions), we intersected across samples with bedtools multiinter, and merged adjacent regions within 1kb with bedtools merge." See lines 284-5.

P-element is italicized here and not elsewhere in the manuscript.

fixed

I don't think it is appropriate to be removing PCR duplicates here? If you can explain why you would do that when its not generally done for RNA-seq.

PCR duplicates were removed from the sequencing of amplicons from genomic DNA, not from the RNAseq data. It was necessary to remove these PCR duplicates to avoid counting them as independent evidence of a new P-element or roo insertion.

Did the methods section used to be after the results section? Figure 1 is what I was looking for in the methods section but its not referenced there, only seeing it in the results. However, the figure is blurry and still a little unclear. Like, what do the blue and red bubbles mean if they aren't used in both figures? If you are indicating P-cytotype with the red bubble, shouldn't it be red on the left hand side too? Or are you trying to indicate something else?

Please see above. We have also exported as a PDF, which hopefully fixes the blurry figure problem (a Mac-PC issue, potentially).

Its weird that the Drosophila/D. contraction is introduced over and over.
Fixed throughout.

By dysgenic you mean not suppressed right? You're calling it the phenotype before you've established it has the phenotype.

We have clarified at the beginning of the results section that we define dysgenic flies as the offspring of a M x P cross with the M-type as the maternal parent, as is standard. We take the reviewers point, though, that we have not yet shown that these are dysgenic, and clarify that these are the flies where we expect to see a lack of P-element regulation and resulting dysgenic phenotypes. Please see paragraph starting at line 306.

We have also substantially rearranged the results section to first show increased P-element expression and transposition rates, establishing a basis for dysgenesis, and then the potential dysgenic phenotypes. We do think this arrangement works much better, and hope the reviewer agrees.

Is it possible that epigenetic differences resulting from maternal inheritance of the Y could be causing these differences? Can you explain why that's not a possibility? Like normally if mom contributes the X you would have mom effects on it, but with the Y? I know Drosophila does not have DNA methylation, but there are plenty of other epigenetic effects.

This is an interesting thought also raised by reviewer 1 and the AE. We hadn't considered previously (as genomic imprinting is generally thought not to occur in flies, despite early reports to the contrary). Please see below where we answer the associate editor's very similar comment.

258 - I feel like no difference in total offspring production is pretty important.....

We agree! The point is that males overall appear to suffer a bit less from P-element activity, and hence the P-element may be able to spread through males more easily. We have hopefully highlighted this rationale more clearly throughout, while still emphasising that this is a hypothesis that our results suggest rather than one that is proven. Please see paragraph starting at line 608.

266 - why define an acronym you never use
Removed

271 - you're calling it the 'effect of dysgenesis' but is it, if they produced the same number of offspring overall?

We have reorganised the results to first establish that the dysgenic F1s show increased P-element activity and possible loss of mature sperm cells, as stated above.

271 -283 - hard to digest this paragraph. How much do these lists overlap? Are the same genes disrupted in any comparisons? This is mentioned once but it's a lot of lists of numbers.

We have substantially revised this section (starting at line 412 and on), which has reduced the complex text in this section. The overlap is specified in Supplementary Figure 4, which we now more clearly reference on line 418.

288 - you defined acronyms in the methods for Molecular Function, biological process etc, but here you are spelling them out. I don't suggest using the acronyms by any means. 285 - 296 -what have we learned or gained from this analysis? Anything? I would argue no.

Overall, we agree that the GO-term analysis adds little insight. We have added a new analysis (see lines 176 and on in Methods and 434 and on in Results), showing that the large numbers of differentially expressed genes between dysgenic and reciprocal flies likely reflect the loss of mature sperm cells. We think this analyses better captures the underlying biology. We keep the GO-term analysis in the main text only to show that the apoptosis genes do not show high levels of expression in dysgenic flies overall, suggesting that any apoptosis expression is transient. We do include the full GO term analysis in the supplement for completeness.

302 - this paragraph seems pretty important. So if you lump old and young together, the expression of the P-element is different, but if you separate them out you no longer have the power to say it is different? Do you have outliers that are reducing your power by a lot? Non-target TEs are significantly differently expressed though. I'm not overly convinced by this analysis so far. I'm confused, maybe I'm misinterpreting what you did. Having the axes all the same might help.
Is there a better way to illustrate the expression and variance in expression of the elements of interest?

We have de-emphasised this result—as the reviewer points out, there isn't a lot of power to detect differential expression. Our main goal here was to see if we could see higher expression in young vs. old flies, consistent with what Khurana et al see for female fertility. We state that we see no evidence of elevated P-element expression in young flies, and leave it at that, with no stronger statement. Please see paragraph at line 352.

In Figure A, its confusing that the P-element would be so close to a log2fold change of maybe 1? But so significant.
The P-element expression is >3-fold higher (log2= 1.78); see line 408, and the p-value adjusted for multiple testing is 0.02. Note that the y-axis is the -log10 of the unadjusted p-value (in the classic volcano plot style). The significance we observed is due to the consistency of this overexpression across samples—it is always overexpressed (> 3-fold) in dysgenic flies, whereas other TEs show more randomness across subsets of the samples.

In the previous version of the plot it was difficult to see the magnitude of the log2-fold difference because of the excessively large x-axis range; we've adjusted this and hope the reviewer finds this an improvement.

315 - so the p-element is differentially expressed in the cross where one might expect it not to change, but not between the crosses where one might expect it to change? Am I getting these results mixed up? **We clarify that the P-element is over-expressed in the expected direction, and the lack of significance in the comparisons involving only young, or only old flies is likely due to the small number of replicates (see lines 348-50). We were primarily looking for the increased regulation in older flies, as suggested by**

Khurana et al, for *D. melanogaster* females, which we saw no evidence of here. We don't make any stronger statement than that due to the low replication of this comparison.

These are a lot of comparisons and genotypes to keep track of, I don't know if there is a clearer way to illustrate the results. Maybe a table.
369 - Ok it seems like this paper is mostly reporting negative results unless I'm mistaken. I don't have an issue with reporting negative results, but more clearly presenting them as such. might be worthwhile.

We have also revamped and reorganised the results section to better highlight the positive results and biological insights. We have also substantially revised this section that was previously around line 369 (see section starting lines 464 and on).

389 - P-element cognate piRNAs and a decrease in P-element mRNA levels - did I miss this being reported? Is this significant? Is there a figure?

The relevant figure is Figure 8B; we added a clearer reference to this figure and report the result statistically starting line 537. We also added a reference to the supplementary figure where the result is plotted in a way that is a little easier to see (but harder to compare to the pattern for TEs overall).

391 - We were also interested in whether TE mRNA and piRNA production from F1 flies resembled an intermediate value between the two parents, or that of the maternal parent Which was it?

We revised this paragraph to eliminate this statement (it turns out that the answer differs by TE, and the original phrasing was not helpful).

401 In contrast to dysgenic F1 flies, there are no large disparities between Shellder mRNA and cognate small RNAs, suggesting that Shellder is well-regulated compared to the P-element (Supplementary Figure 5C). On what basis are we saying that the P-element isn't well regulated?

We hope that the restructuring of the manuscript better presents the evidence that the P-element is poorly regulated in dysgenic flies better—expression is higher, and transposition is higher for the P-element in these flies.

436 - I don't understand the caveat? Do you mean there was dysgenesis in flies that didn't differ in p-element status?
We simplified and revised this section. Essentially, we were concerned that the crosses used to produce the sequenced F2s were in two of four cases dysgenic and may have added some transpositions to our data. However, the results don't really bear this worry out—there is no statistical difference between the dysgenic and non-dysgenic F2 crosses. This is as these crosses were performed at 25ºC, where P-element activity is lower, and only transposition in early embryogenesis in the F2s would be likely to show up in our data.
This caveat thus doesn't bear on our main result that dysgenic F1s show higher transposition rates.

None of this considers the fact that the P-element has considerable evidence for regulation by alternative splicing as well as piRNA. I know this is often disregarded in manuscripts on the P-element. "Unexpectedly, we show that the piRNA pathway components do not act to reduce transcript levels of the P-element transposon during P-M hybrid dysgenesis, a syndrome that affects germline development in Drosophila. Instead, splicing regulation is mechanistically achieved together with piRNA-mediated changes to repressive chromatin states, and relies on the function of the Piwi-piRNA complex proteins Asterix (also known as Gtsf1) and Panoramix (Silencio), as well as Heterochromatin protein 1a (HP1a; encoded by Su(var)205). "

This is a very good point, and we are grateful to the reviewer for pointing out. We now show that the splicing rate is higher in dysgenic flies compared to controls; see lines (see paragraph starting line 352).

Associate Editor Comments:

I thought your use of a compound-X in your crossing scheme, producing males with identical chromosomal genotypes but different cytotypes, was a very clever approach. However, it does mean that the parent-of-origin for the sex chromosomes is reversed (e.g. the Y is matroclinous in the cross with a compound-X mother, and patroclinous in the cross with a normal-X mother.)

We definitely needed to make this clear, and hope we have now done so at the beginning of the Results (see paragraph starting line 306).

We agree that attached-X crosses are clever, but the cleverness belongs to fly genetics not us, or rather specifically to Lillian Morgan, whom we now cite.

Do you think that this parent of origin difference could lead to epigenetic changes that might be having an effect? I recognize that this difference would also be confounded with the cytotype differences in this particular cross, but a brief discussion of this point (with possible experiments that might tease these apart) would be welcome.

This is a very interesting point that sent us down a bit of a rabbit hole (enjoy : /).

As attached-X strains are frequently used to control sex-chromosome inheritance exactly as we have done here (e.g., doi:10.1093/molbev/msaa082 , doi:10.1093/molbev/msad152) , we hadn't really considered this point before—rather, we thought of this as a standard method that didn't require a lot of thought or justification.

HOWEVER, Lemos et al. (https://doi.org/10.1534/g3.113.008029) look at exactly this effect in both males in females in *D. melanogaster* in crosses involving attached-X stocks. Using microarrays, they find similar levels of differentially expressed genes (~1000) in males with different Y-chromosome origins, as we do here, and point out that the effect is especially strong in testes.

BUT, we think Lemos et al. are also looking at a dysgenic vs. reciprocal control crosses, though they didn't consider this possibility.

Specifically, the AutW132 strain used in this experiment appears in the literature around ~2010 (mostly used by David Rand's lab and trainees) and is identified as a wild-type strain caught in Austria by Christian Schlotterer (https://doi.org/10.1101/231852). This is almost certainly a P-type strain, as Christian's career spans the early 1990s-present, when the P-element was already widespread in *D. melanogaster*. The attached-X used is a from an old lab strain, introgressed onto the AutW132 background, but through crosses involving lab females, and thus almost certainly M-type.

Thus, the males that they compare for their differentially expression analysis differ both in Y-parent of origin and in cytotype, exactly as do ours. Like us, they can't separate parent of origin effects from cytoplasmic ones-- as they say themselves. Of note, the females they compared both have AutW132 (likely P-cytotype) mothers, and show few differences in gene expression.

They do argue that these are not the same cytoplasmic effects as seen in Innocenti et al. (10.1126/science.1201157; where it appears P-cytotype was introgressed into an M-type background; doi: 10.1111/j.1474-9726.2008.00428.x.). But that doesn't really bear on the whether this different effect is due to parent-of-origin effects or something else, such as dysgenesis. (We also weren't super convinced by this argument, as it seems either way there are massive changes in gene expression…)

We feel dysgenesis provides a simpler explanation than parent-of-origin effects for these very similar results for two reasons: i) P-element dysgenesis in the germline, where they observe the largest effects, is well-established ii) the large shifts in gene expression are more parsimoniously explained by a loss of mature germ cells than by thousands of independent regulatory effects on genes effected by a maternal origin of the Y-chromosome, iii) dysgenic effects, though more extreme at 29ºC, occur at standard temperatures (including sterility in females), and so would also have affected their results.

We discuss these issues in brief on lines 896 and on, hopefully gracefully.

July 24, 2026
RE: GENETICS-2026-309639

Prof. Andrea Betancourt
University of Liverpool
Evolution, Ecology, and Behaviour
Crown St.
Biosciences Building
Liverpool L69 7ZB
United Kingdom

Dear Dr. Betancourt:

Congratulations, your manuscript titled "The impact of P-Element-induced hybrid dysgenesis on the male germ-line in Drosophila simulans" is accepted for publication in GENETICS! Many thanks for submitting your research to the journal.

The reviewers had a couple of suggestions for improving the manuscript that you may want to consider. You can view their comments at the bottom of this email. I would specifically suggest you address Reviewer 1's comment concerning figure 6 (the "psi" genes and the gene called "sterile") as well as Reviewer 2's comment about describing your use of roo elements as a control.

To Proceed to Publication:
1. Format your article according to GENETICS style: https://academic.oup.com/genetics/pages/author-guidelines

2. Ensure that you comply with data and community resource citation guidelines:
https://academic.oup.com/genetics/pages/author-guidelines#section-5-9-2

3. Upload your final files at https://genetics.msubmit.net

4. Add oupsupport@scipris.com and genetics.oup@novatechset.com (or the domains @scipris.com and @novatechset.com) to your email program's "safe senders" list. You will be contacted by both at various points during the production process.

Notes:
- Your currently-accepted manuscript (unedited, as submitted, reviewed, and accepted) will be published at GENETICS and deposited into PubMed as an Advance Access article. Notify sourcefiles@thegsajournals.org before signing your license if you do not wish to publish your article via Advance Access.

- We invite you to submit an original color figure related to your paper for consideration as cover art. Please note that submissions created with AI image generation tools are not eligible. Please email your submission to the editorial office or upload it with your final files. You can submit a small-sized image for evaluation, and if selected, the final image must be a TIFF file 2513px wide by 3263px high (8.375 by 10.875 inches; resolution of 600ppi). Please avoid graphs and small type.

- After files are sent to Oxford University Press we use SciPris to manage article licensing and payment. If you do not have a SciPris account, you will receive an email from no-reply@scipris.com to sign up to use Oxford University Press' author portal. After logging in, follow the online instructions to sign your license and arrange any payment due.

If you have any questions or encounter any problems while uploading your accepted manuscript files, please email the editorial office at sourcefiles@thegsajournals.org.

Sincerely,

William Gilliland
Associate Editor
GENETICS

Approved by:
Amy MacQueen
Senior Editor
GENETICS

Review comments (if applicable):

Reviewer #1 :

For the p-value in figure 2B, was this estimated by simply binning old and young samples as biological replicates? I am fine with that, but I wasn't able to find a clear explanation in: 2B Figure legend, Lines 324 to 331 or 194 to 196. Some clarity would be helpful.

what are the Psi genes in figure 6? And the gene "sterile"? I have never heard of them.

I know it is probably annoying for a reviewer to raise an issue in the second review, but this is still based in the challenge I have in interpreting 9A. It is hard to wrap my head around 9A and only after reading this version was I able to digest it properly since this figure is not labeled by TE family and contains many different conditions and they opted not to parse it out per my previous suggestion (which is fine) Also, as far as I can tell from the tracked changes document, the text on lines 538 to 540 are new.

" negative (Pearson's r = -0.915, p = 0.0292; Figure 8B). Consistent with a failure of P-element regulation by piRNAs, dysgenic individuals had the highest ratio of P-element mRNAs to piRNAs (excepting the M-type attached-X stock), mainly due to higher levels of mRNA (Supplementary Figure 8C)."

The result in 9A arises from a combination of all TE families, but 9B is for a single TE family. I believe the proper analysis should include a measure of the distribution of correlation coefficients for each TE family individually (as in 9B) and a comparison should be made accordingly with respect to either the slope or the R^2 value. Also, can you explain why the R^2 value here is negative? Is it because the intercept was forced? Having the results of 9A and 9B are interesting, but I think they should still dig in a bit more on the statistics.

Reviewer #2 :

I find the manuscript to be considerably improved after revision. In particular, I appreciate the authors willingness to look at splicing of the P-element. The analysis was very interesting and well presented. I would suggest one minor revision - line 254 is the first place that roo is mentioned, without a clear explanation of why it is being mentioned. If you could add a sentence explaining that it is the control, that would help with the overall clarity of the manuscript.

Typos:

Line 579: "dimophism"
Supplementary Table 4 Legend, line 6: "encodingg"
