# Supplementary material for: The impact of P-element-induced hybrid dysgenesis on the male germline in Drosophila simulans
Source: Genetics. 2026 Aug 20;234(2):iyag219. doi: 10.1093/genetics/iyag219 (PMC13643802; doi:10.1093/genetics/iyag219)
Supplement: Supplementary file 2 [file iyag219_supplementary_data.zip › Supplementary_Figures_GENETICS-2026-309639.docx]

**Supplementary Figure 1.** Library preparation protocol to sequence breakpoints of TE insertions, following Grech et al (2019). Linker and primer sequences are given in Supplementary Table 2. The initial input for library preparation was genomic DNA isolated from ~8 flies and then pooled, as described in the Materials and Methods.

*End-repair and A-tailing* was performed for each of the 286 pools of ~eight flies.

*Linker ligation* was also performed for each of the 286 pools of ~eight flies. We ligated a forked linker, composed of Linker-1 and Linker-2. Linker-1 contains a 6nt barcode for pool identification, and a random 4-mer. Linker-2 contains a modified base on the 3’ end that prevents linker-linker amplification in the first PCR cycle (see below).

*Pooling.* Following linker ligation, the barcodes in Linker-1 can be used to identify the specific original pool of ~eight flies (“small pool”). Barcoded “small pools” were subsequently pooled into six “large pools”, such that each “large pool” is formed of ~48 “small pools” (or approximately 8 x 48 = 384 flies). Subsequent PCR steps were performed on “large pools”.

*PCR1.* In the first round of PCR, the ends of TEs and adjacent genomic regions were selectively amplified. Two primers were used, a TE-primer partly complementary to part of the TE of interest (P-element or roo), and Linker1-amp, a primer complementary to the unique part of Linker 1 (not present in Linker 2). In the initial cycle of this first PCR, extension occurred only from the TE primer (due to the 3’ blocker on Linker 2), which forms the binding site for the Linker1-amp. In subsequent cycles, both primers can then anneal and be extended, and both strands are amplified.

*PCR2.* In the second round of PCR, standard Illumina primers (NEBNext Multiplex Oligos for Illumina) are used to create barcoded libraries for sequencing (each library corresponding to one “large pool” and TE target).

This library preparation protocol was performed separately for P-element and roo, for a total of 12 sequenced libraries (6 ‘large pools’ for each TE).

**Supplementary Figure 2.** The effect of dysgenesis on TE expression in testes. Top row comparisons are between A) old, and B) young dysgenic F1 vs. reciprocal control males (BEL_LTR refers to long-terminal repeats of BEL, and not the full element).

The middle row shows the P-type parent (Cro18) vs. C) reciprocal and D) dysgenic F1s, and the bottom row shows the M-type, attached-X parent vs. E) reciprocal and F) dysgenic F1s. TEs with adjusted p-values < 0.05 are shown in orange, those with adjusted p-values < 0.05 and a log-2-fold-change > 1 are show in red. The P-element is labelled regardless of significance.

**Supplementary Figure 3.** Principal component analysis coordinate plot showing normalised counts of transcripts from DESeq2, obtained from RNA-seq data quantified by Salmon.

**Supplementary Figure 4.**  Effect of dysgenesis on global gene regulation in testes from young vs. old flies. Volcano plots show comparison between A) only old dysgenic and reciprocal flies, and B) only young dysgenic and reciprocal flies. Genes with adjusted p-values < 0.05 are shown in orange, those with adjusted p-values < 0.05 and a log-2-fold-change > 1 are show in red.

The Venn diagrams show the number of genes with C) higher, and D) lower expression in dysgenic F1 vs. reciprocal controls, with comparisons done for the young and old age classes separately. Acronyms refer to hybrid dysgenic young (HDY), reciprocal young (RY) and hybrid dysgenic old (HDO) and reciprocal old (RO) samples. ‘Down’ and ‘up’ indicate whether the gene was down- or up-regulated in the first sample of the comparison (e.g., ‘Down_HDO vs RO’ indicates genes that were down-regulated in HDO compared to RO testes).

**Supplementary Figure 5.** Gene ontology (GO) term over-representation analysis of genes differentially expressed between dysgenic and reciprocal testes shared among the old and young samples. The top 10 most significant GO terms are shown.

**Supplementary Figure 6.** Ping pong signatures shown by an enrichment of sense and anti-sense piRNAs with a 10-bp overlap for piRNAs cognate to A) all TEs, and B) the P-element. Note that Z-scores > 2 indicate a significant enrichment at the alpha = 0.05 level. There were no overlapping P-element piRNAs in attached-X flies (XX), and few in the reciprocal young sample; all others show z-scores > 2 for overlaps of 10 nucleotides.

**Supplementary Figure 7.** Expression of piRNAs expressed in testes from dysgenic vs. reciprocal males in different age classes. Shown are A) old testes dysgenic vs. reciprocal samples; and B) young testes dysgenic vs. reciprocal samples. Labels show names of cognate TEs; those with adjusted p-values < 0.05 and a log-2-fold-change > 1 are show in red.

**Supplementary Figure 8.**  Principle components analyses of TPM values from SalmonTE on TE expression levels (A), piRNA expression levels (B) and bi-plots showing the major contributors to differences on PC1 (the P-element; P_Dsim) and PC2 (Shellder) for both TE mRNA (C) and piRNAs (D). Expression levels of P-element (E) and Shellder (F) in the different samples are shown.
