# Supplementary material for: The impact of P-element-induced hybrid dysgenesis on the male germline in Drosophila simulans
Source: Genetics. 2026 Aug 20;234(2):iyag219. doi: 10.1093/genetics/iyag219 (PMC13643802; doi:10.1093/genetics/iyag219)
Supplement: Supplementary file 2 [file iyag219_supplementary_data.zip › Supplementary_Tables_GENETICS-2026-309639.pdf]

Supplementary Table 1. Fly crosses and pooling strategy for transposition assays, used to measure transposition in the germline of dysgenic (HD) and reciprocal control (R) F1 males and females. Libraries targeting specific TEs (*P*-element and *roo*) were prepared from F2 flies produced as detailed below. Library indicates the type of individual targeted for transposition measurement. Pooled F2s are the sequenced flies. Crosses in bold are M-female x P-male crosses that may induce dysgenesis.

**Library**	**Initial cross (28.5°C)**	**Second cross (25°C)**	**Pools**		
			**sex**	**pools**	**flies/ pool**
Cro18	-	-	F	1	8
	-	-	M	1	8
M252	-	-	F	1	8
XX	-	-	Mixed	1	8
HD-♀	**M252 ♀ x Cro18 ♂**	F1♀ x M252♂	F	59	472
R-♀	Cro18 ♀ x M252 ♂	F1♀ x M252♂	F	61	488
HD-♂	**XX ♀ x Cro18 ♂**	**F1♂ x XX♀**	F	29	232
			M	41	328
			Mixed	1	6
				total	*566*
R-♂	Cro18 ♀ x XX ♂	**F1♂ x XX♀**	F	19	152
			M	51	410
			Mixed	1	8
				total	*570*

Supplementary Table 2. Oligos used in library preparation to identify TE insertion locations.

Primer name	Sequence	Notes	purification
Linker 1	TTCAGACGTGTGCTCTTCCGATCTXXXXXXNNNN**CTCCGCT** **TAAGGGAC*T**	Sample-specific; contains an index to identify the sample (XXXXXX) + a random 4-mer (NNNN); bold sequence is complementary to Linker 2	HPLC
Linker 2	/5Phos/GTCCCTTAAGCGGAG/3AmMO	phosphate group on 5' end; amino group in its 3' end	desalted
Linker 1-Amp	TTCAGACGTGTGCTCTTCCGATCT	same as underlined portion of Linker 1	HPLC
Pelement-R	CACGACGCTCTTCCGATCTNNNNTCGTCCGCACACAACCTT TC	red sequence is the same as part of an Illumina universal primer used in PCR2; NNNN is a random 4-mer, blue sequence is complementary to the TE	desalted
Roo-R	CACGACGCTCTTCCGATCTNNNNCGATTCATTTACATAAGA TATGAACGGAGCC		desalted
Universal primer	AATGATACGGCGACCACCGAGATCTACACTCTTTCCCTACA CGACGCTCTTCCGATCT	contains part of the TE-specific primer (red sequence)	HPLC
Primer-Index	CAAGCAGAAGACGGCATACGAGATCGTGATGTGAZZZZZZT TCAGACGTGTGCTCTTCCGATC*T	underlined region also in linker 1; contains a 6nt index (ZZZZZZ) to barcode the library; * is a phosphorothioate bond	HPLC

Supplementary Table 3. Characteristics of the RNAseq samples from testes used in this study. Mapping was done as described in the main text, and mapping rates are from Salmon or SalmonTE as appropriate. Sample names indicate whether the sample is from a parental strain (P-type Cro18 or M-type attached-X), or from an F1 and in which direction (HD = hybrid dysgenic, with an attached-X maternal parent, R = reciprocal, with a Cro18 maternal parent). Numbers are used to separate replicates.

Cross direction	Age	Sample	Library size (n read pairs)	Mapping rate: transcriptome	Mapping rate: TEs
**Parent**	Young	20-Cro18	78,877,802	**71.35%**	0.5268 %
**Parent**	Young	30-Cro18	76,828,078	72.07	0.5493
**Parent**	Young	20-XX	69,919,986	87.01	0.6673
**Parent**	Young	30-XX	85,535,342	86.77	0.6835
**Dysgenic**	Young	5-HDY	85,556,986	83.46	0.5491
**Dysgenic**	Young	6-HDY	87,834,072	85.80	0.4774
**Dysgenic**	Old	7-HDO	69,779,714	82.87	0.8228
**Dysgenic**	Old	8-HDO	97,114,280	82.40	1.0258
**Reciprocal**	Young	9-RY	78,278,758	60.11	0.2772
**Reciprocal**	Young	10-RY	60,430,212	65.74	0.3541
**Reciprocal**	Old	11-RO	93,116,912	71.45	0.4289
**Reciprocal**	Old	12-RO	73,876,468	75.56	0.7183

Supplementary Table 4. Estimates of the number of transcripts corresponding to splice variants of the P-element, resulting in transcripts encoding transposase (IVS3 spliced; coordinates in Genbank accession X06779: 1947-2138) or one of two alternatively spliced transcripts encoding a truncated protein. Estimates of splicing of this intron are STAR as described in the main text. IVS3 stands for intervening sequence 3, or the third intron of the P-element transcript. In IV3 spliced transcripts, this intron is removed fully. IV3.1 and IV3.2 are subsequences of this intron that spliced from alternate donor (GT-) and acceptor sites (-AG), resulting in the retention of a small exon (at 2015-2049), resulting in a transcript encodingg a truncated transposase; the 'spliced non-functional' column contains the estimated number of transcripts containing this exon from the average of IV3.1 and IVS3.2 splice estimates. The columns Exon2-IVS3 and IVS3-Exon3 junctions are obtained from reads covering the intron-exon junctions from samtools depth, and are averaged to estimate the number of unspliced transcripts. The sum of spliced non-functional and unspliced transcripts is summed for the total number of non-functional transcripts. Numbers in bold are those used for statistical tests of functional *vs* nonfunctional transcripts for each sample type.

	**Coding transcript**	**Transcripts encoding non-functional transposase**						
**Sample**	**IVS3 spliced**	**IVS3.1**	**IVS3.2**	**spliced non-functional**	**Exon2-IVS3 junction**	**IVS3-Exon3 junction**	**fully unspliced**	**All non-functional**
P-type (Cro18)	**0**	3	1	2	28	46	37	**39**
P-type (Cro18)	**0**	2	1	2	32	25	28.5	**30**
Hybrid Dysgenic Old	**8**	83	76	80	127	146	136.5	**216**
Hybrid Dysgenic Old	**5**	55	50	53	162	169	165.5	**218**
Hybrid Dysgenic Young	**12**	62	64	63	114	177	145.5	**209**
Hybrid Dysgenic Young	**7**	52	49	51	130	142	136	**187**
Reciprocal Old	**0**	20	18	19	31	43	37	**56**
Reciprocal Old	**0**	8	7	8	37	35	36	**44**
Reciprocal Young	**0**	3	4	4	29	30	29.5	**33**
Reciprocal Young	**0**	11	6	9	26	23	24.5	**33**
M-type (attached X)	**0**	0	0	0	0	0	0	**0**
M-type (attached X)	**0**	0	0	0	0	0	0	**0**

Supplementary Table 5. Significant GO terms for MF and CC in the 112 upregulated DEGS for dysgenic vs. reciprocal flies that were common to the testes of young and old flies.

	GO.ID	Term	Annotated	Significant	Expected	classicFisher
Molecular Function	GO:0050839	cell adhesion molecule binding	17	3	0.11	0.00016
	GO:1901363	heterocyclic compound binding	786	11	5.05	0.0079
Cellular Component	GO:0045178	basal part of cell	35	3	0.2	0.00099
	GO:0098858	actin-based cell projection	12	2	0.07	0.00203
	GO:0098590	plasma membrane region	124	4	0.71	0.00516
	GO:0009925	basal plasma membrane	25	2	0.14	0.00881

Supplementary Table 6. Significant GO terms for BF, MF and CC in the 104 downregulated DEGS for dysgenic vs. reciprocal flies that were common to the testes of young and old flies.

	**GO.ID**	**Term**	**Annotated**	**Significant**	**Expected**	**classicFisher**
Biological Function	GO:0000413	protein peptidyl-prolyl isomerization	16	2	0.04	0.00085
	GO:0018208	peptidyl-proline modification	16	2	0.04	0.00085
Molecular Function	GO:0003755	peptidyl-prolyl cis-trans isomerase acti...	27	2	0.06	0.0016
	GO:0016859	cis-trans isomerase activity	29	2	0.07	0.0019
Cellular Component	GO:0016020	membrane	2543	12	7.06	0.0098

Supplementary Table 7 Characteristics of the small RNAseq samples from testes used in this study. Filtering and mapping were done as described in the main text. Sample names indicate whether the sample is from a parental strain (P-type Cro18 or M-type attached-X), or from an F1 and in which direction (HD = hybrid dysgenic, with an attached-X maternal parent, R = reciprocal, with a Cro18 maternal parent). Numbers are used to separate replicates.

Cross direction	Age	Sample	Library size pre-filtering	Library size post-filtering	SalmonTE mapping rate
**Parent**	Young	13-Cro-4	20,336,238	7,295,922	0.1544 %
**Parent**	Young	14-Cro-5	22,489,472	8,020,712	0.1707
**Parent**	Young	15-XX-4	18,617,008	6,550,881	0.1563
**Parent**	Young	16-XX-5	61,032,013	22,239,761	0.1305
**Dysgenic**	Young	17-HDY	9,727,359	3,227,352	0.1082
**Dysgenic**	Young	18-HDY	10,252,686	3,876,066	0.1700
**Dysgenic**	Old	19-HDO	4,086,151	1,723,350	0.1980
**Dysgenic**	Old	20-HDO	87,242,240	39,386,956	0.2314
**Reciprocal**	Young	21-RY	31,472,367	12,916,110	0.1496
**Reciprocal**	Young	22-RY	65,356,370	25,511,053	0.1161
**Reciprocal**	Old	23-RO	1,122,033	396,804	0.1285
**Reciprocal**	Old	24-RO	16,417,402	5,420,735	0.1409
